# Monoclonal Gammopathy of Undetermined Significance and Associated Cardiovascular Outcomes in a Hospital Setting—A Fresh Perspective †

**DOI:** 10.3390/curroncol31080331

**Published:** 2024-08-01

**Authors:** Ahmad Mustafa, Chapman Wei, Ghada Araji, Muhammad Rafay Khan Niazi, Radu Grovu, Mitchell Weinberg, James Lafferty

**Affiliations:** 1 Department of Cardiology, Northwell Health/Staten Island University Hospital, Staten Island, New York, NY 10305, USA; mweinberg4@northwell.edu (M.W.); jlafferty1@northwell.edu (J.L.); 2 Department of Internal Medicine, Northwell Health/Staten Island University Hospital, Staten Island, New York, NY 10305, USA; cwei4@northwell.edu (C.W.); rgrovu@northwell.edu (R.G.); 3 Department of Hematology and Oncology, Northwell Health/Staten Island University Hospital, Staten Island, New York, NY 10305, USA; mniazi@northwell.edu

**Keywords:** MGUS, cardiovascular outcomes, cardiovascular comorbidities, non-cardiovascular comorbidities, cardiovascular diseases

## Abstract

There is a paucity of data on the cardiovascular implications of monoclonal gammopathy of undetermined significance, especially among hospitalized patients. Our study aimed to investigate the association between MGUS and cardiovascular outcomes in a hospital setting using the National Inpatient Sample database. MGUS patients were sampled using ICD-10 codes. The patients were stratified into two cohorts based on the presence or absence of MGUS. Comorbidities and cardiovascular outcomes were collected using ICD 10 DM codes. CV outcomes were evaluated before and after 1:1 matching for age, gender, and race. Furthermore, a sensitivity analysis was performed on the matched population, which excluded patients with diabetes mellitus, prior myocardial infarction, chronic kidney disease (stages 3–5), dialysis, hypertension, obesity, metabolic syndrome, cancer, antiplatelets, and oral anticoagulant use and was adjusted for smoking, dyslipidemia, and aspirin use to evaluate the cardiovascular outcomes. MGUS patients had more heart failure, atrial fibrillation, venous thromboembolism, aortic aneurysm, aortic stenosis, aortic regurgitation, mitral stenosis, mitral regurgitation, conduction disorder, cor pulmonale, peripheral vascular disease, and acute myocardial infarction. After matching, MGUS was associated with heart failure, atrial fibrillation, venous thromboembolism, aortic stenosis, mitral regurgitation, conduction disorder, cor pulmonale, and peripheral vascular disease. MGUS was linked to a wide spectrum of cardiovascular diseases in an inpatient setting. Further studies are needed to formulate appropriate recommendations for the screening and management of cardiovascular complications in individuals with MGUS.

## 1. Introduction

Monoclonal gammopathy of undetermined significance (MGUS) is one of the most prevalent asymptomatic premalignant conditions associated with a risk of malignant transformation into multiple myeloma (MM) or other forms of lymphoproliferative disorders [1]. MGUS diagnosis requires the presence of <3 g/dL of serum M-protein and <10% bone marrow plasma cells, with no evidence of myeloma-related end-organ damage (hypercalcemia, renal insufficiency, anemia, or bone lesions) [2]. MGUS is further classified into three subtypes that include (1) non-IgM MGUS (IgG, IgA, or IgD MGUS), the most common subtype with an increased propensity to progress to MM, solitary plasmacytoma, and AL amyloidosis; (2) IgM MGUS, which accounts for approximately 15% of cases, with an increased propensity to progress to Waldenstrom macroglobulinemia, AL amyloidosis, and, rarely, IgM MM; and (3) light-chain MGUS, a subtype in which secreted M-proteins lack the heavy-chain component of immunoglobulin, with a risk of progression to light-chain MM and AL amyloidosis [3,4]. The incidence of MGUS increases with age, with a prevalence of 1–2% in adults older than 50 years of age and up to 5.3% in patients older than 70 years of age [5]. Although it is benign and premalignant, a growing body of evidence has confirmed the association between MGUS and a variety of diseases, including bone disease, renal impairment, autoimmune disorders, neuropathies, secondary immunodeficiencies, and infections [6,7].

Recently, there has been an emerging focus on cardiovascular (CV) outcomes in patients with MGUS, and a new term, “monoclonal gammopathy of undetermined cardiovascular significance”, has been proposed [8]. The CV mortality rates in MGUS patients are higher compared with other causes of mortality [9,10]. Moreover, venous thromboembolism (VTE) and arterial thrombosis have also been reported to be more frequent in MGUS patients [11,12,13,14,15,16]. However, our understanding of the CV risk factors and underlying pathogenesis in MGUS remains largely limited. Several hypotheses have been proposed to explain these associations. For example, increased levels of pro-inflammatory cytokines (interleukin-6) and tumor necrosis factor upregulate the transcription of von Willebrand factor and fibrinogen in MGUS patients, which may contribute to the increased risk of arterial thrombosis [17]. More recently, clonal hematopoiesis of indeterminate potential (CHIP), which may coexist with MGUS, has been implicated in atherosclerotic arterial disease and the production of pro-angiogenic cytokines [18,19,20,21]. Recently, a Danish study investigated the association of MGUS with a spectrum of CV outcomes. MGUS patients had an increased risk of developing a wide range of cardiovascular diseases, including heart failure, ischemic heart disease, valvular disease, conduction abnormalities, VTE, and peripheral arterial disease, even after adjusting for common comorbidities [22].

There is a paucity of data regarding the inpatient health outcomes of MGUS patients as they are more likely to present in an outpatient setting rather than a hospital, given MGUS’s typical asymptomatic presentation. Only about 0.1–14% of MGUS patients, globally, are admitted to a hospital [23,24,25]. Studies that reported on patient outcomes did not extensively evaluate cardiovascular outcomes and were primarily conducted to estimate the frequency of MGUS patients being admitted to a hospital. Our current study investigated the association of MGUS with cardiovascular outcomes in a hospital setting.

## 2. Methods

Patients were recruited from the National Inpatient Sample (NIS) database (2016–2018). The NIS database is the largest inpatient database in the United States funded through the Healthcare Cost and Utilization Project (HCUP) [26]. The IRB is exempt as per the HCUP data use agreement since patient information is de-identified.

Demographics and comorbidities were sampled using the International Classification of Disease 10 codes. The patients were stratified into two cohorts based on the presence or absence of MGUS. MGUS patients were defined by having serum M-protein levels of <3 g/dL and <10% bone marrow plasma cells, with no evidence of myeloma-related end-organ damage. Patients aged less than 18 years or those with missing data were excluded. Additionally, 4,054,809 patients with concomitant multiple myeloma, smoldering multiple myeloma, B-cell lymphomas, non-Hodgkin lymphomas, amyloidosis, and Waldenstrom’s macroglobulinemia were excluded.

### 2.1. Comorbidities

Comorbidities included hypertension, diabetes mellitus (DM), smoking, chronic kidney disease (CKD), dialysis dependence, obesity, dyslipidemia, metabolic syndrome, all-cause cancer, aspirin use, antiplatelet use, and oral anticoagulant use.

### 2.2. Cardiovascular Outcomes

The CV outcomes evaluated included heart failure, atrial fibrillation, acute myocardial infarction (MI), aortic aneurysm, aortic dissection, aortic stenosis, aortic regurgitation, mitral stenosis, mitral regurgitation, conduction disorders, pericarditis, peripheral vascular disease (PVD), cor pulmonale, VTE, and ischemic stroke/transient ischemic attack (TIA).

### 2.3. Statistical Analysis

Initially, univariate analysis was conducted to assess the CV outcomes between the two groups. Greedy propensity matching was then performed using R version 4.1.2 (R Foundation for Statistical Computing, Vienna, Austria). MGUS and non-MGUS patients were matched 1:1 for age, gender, and race. The caliper for the propensity score difference was 0.0000001. After propensity matching, the mean difference in propensity scores was less than 0.1 for age, gender, and race, which signified successful matching. Furthermore, chi-square analyses confirmed that the two groups were similar in terms of age, gender, and race (*p* = 1.000). The 2 cohorts were then compared for the above-mentioned cardiovascular outcomes. *p*-values of less than 0.05 were considered statistically significant.

Furthermore, a sensitivity analysis was performed. All individuals with a history of DM, prior MI, hypertension, CKD (stages 3–5), dialysis dependence, obesity, metabolic syndrome, cancer (all-cause), antiplatelet, or oral anticoagulant use were excluded. The CV outcomes were then re-evaluated using multivariate logistic regression to adjust for smoking, dyslipidemia, and aspirin use.

Continuous variables were analyzed using Student’s *t*-tests and ANOVAs. Categorical variables were analyzed using chi-square analyses and Fisher’s exact tests. All statistical analyses were performed using IBM SPSS Statistics for Windows, version 28 (IBM Corp., Armonk, NY, USA) and R version 4.1.2 (R Foundation for Statistical Computing, Vienna, Austria).

## 3. Results

Out of a total of 17,357,556 patients included in this study, 23,435 patients had MGUS (0.1%). MGUS patients were older, predominantly male, smokers, and Caucasian. Additionally, MGUS patients had more DM (39.2% vs. 27.9%), CKD (33.6% vs. 8.6%), dialysis dependence (13.3% vs. 4%), obesity (18% vs. 16.5%), dyslipidemia (39.1% vs. 23.3%), metabolic syndrome (18.9% vs. 10.4%), and cancer (12.9% vs. 8.1%) when compared with non-MGUS patients (Table 1). The cancer subtypes in both cohorts are listed in Supplementary Table S1 and show a similar distribution, apart from prostate and hematologic cancers, which were more prevalent in MGUS patients. Aspirin, antiplatelet, and anticoagulant use were more prevalent in MGUS patients compared with non-MGUS patients. The most prevalent non-cardiovascular comorbidity was DM (39.2%).

The univariate analysis showed that MGUS patients had more heart failure (36.8% vs. 17.2%; *p* < 0.001), atrial fibrillation (10% vs. 5.9%; *p* < 0.001), acute MI (4.5% vs. 3.6%; *p* < 0.001), aortic aneurysm (2.2% vs. 1%; *p* = 0.005), aortic stenosis (4.0% vs. 1.6%; *p* < 0.001), aortic regurgitation (1.2% vs. 0.5%; *p* < 0.001), mitral stenosis (0.3% vs. 0.1%; *p* = 0.009), mitral regurgitation (3.5% vs. 1.5%; *p* < 0.001), conduction disorder (6.6% vs. 3.2%; *p* = 0.002), PVD (14.4% vs. 5.6%; *p* < 0.001), cor pulmonale (0.4% vs. 0.2%; *p* < 0.001), VTE (2.8% vs. 1.6%; *p* < 0.001). Acute MI (4.5% vs. 5.3%; *p* < 0.001), and stroke/TIA rates (2.8% vs. 2.6%; *p* < 0.001). There were no significant differences in ischemic stroke/TIA and aortic dissection rates between both groups (Table 2).

**Figure 1:**
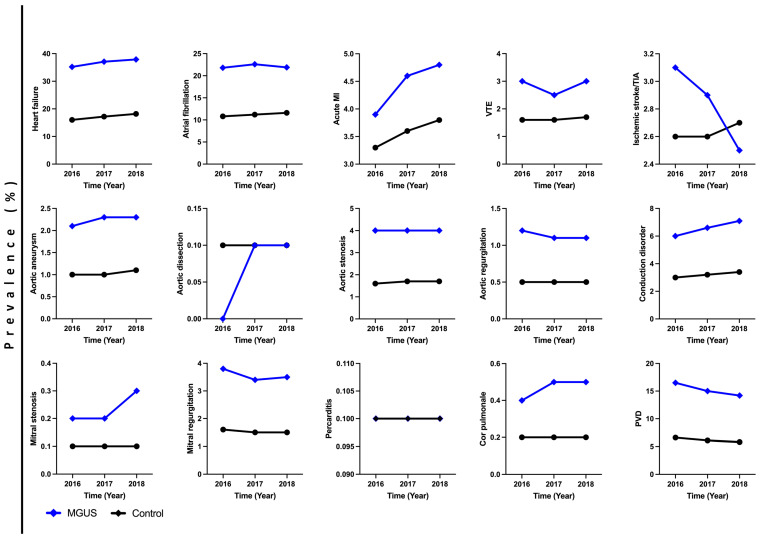
Prevalence of cardiovascular outcomes in MGUS vs. matched controls from 2016 to 2018. MI—myocardial infarction; VTE—venous thromboembolism; TIA—transient ischemic attack; PVD—peripheral vascular disease.

We trended the cardiovascular comorbidities and outcomes from 2016 to 2018 (Figure 1). In MGUS patients, there was an increase in the prevalence of heart failure, acute MI, aortic aneurysm, mitral stenosis, conduction disorder, and cor pulmonale rates from 2016 to 2018. There was a decrease in ischemic stroke/TIA, aortic regurgitation, mitral regurgitation, and PVD rates from 2016 to 2018.

After matching the two cohorts for age, gender, and race, our matched model corroborated several of our unmatched findings indicating that MGUS was associated with higher heart failure, atrial fibrillation, VTE, aortic stenosis, mitral regurgitation, conduction disorder, cor pulmonale, and PVD (Table 3). Acute MI and ischemic stroke/TIA rates were negatively associated with MGUS in our matched models.

Finally, sensitivity analysis was performed, which excluded patients with DM, hypertension, prior MI, CKD (stages 3–5), dialysis dependence, obesity, metabolic syndrome, cancer (all-cause), smoking, dyslipidemia, and aspirin, antiplatelet, and oral anticoagulant use (Table 3). In this sub-group population, MGUS was still associated with higher rates of heart failure, atrial fibrillation, VTE, aortic stenosis, aortic regurgitation, mitral regurgitation, cor pulmonale, and PVD. There were no significant differences in acute MI and ischemic stroke/TIA rates between both groups in this sub-group analysis.

## 4. Discussion

In our large current retrospective study, we comprehensively evaluated the association of MGUS with cardiovascular comorbidities and outcomes in hospital settings. For matched cohorts, MGUS was associated with increased heart failure, atrial fibrillation, VTE, aortic stenosis, mitral regurgitation, conduction disorders, cor pulmonale, and peripheral vascular disease relative to non-MGUS patients. In the analyses, however, MGUS was found to be less associated with acute MI. MGUS patients without significant non-cardiovascular comorbidities showed similar associations.

MGUS patients had a statistically significant increased risk of VTE. Multiple cohort studies have shown a significantly increased risk of VTE in MGUS patients [11,12,14,16,22,27]. One study did not show an increased risk of VTE, probably due to the small population size (166 patients) [15]. Interestingly, the risk of thrombosis varies within various types of MGUS depending on immunoglobulins. One study showed that patients with IgM MGUS did not have an increased risk of thrombosis, whereas patients with IgG/IgA MGUS had a four-fold increased risk of venous thrombosis [11]. On the contrary, in a different study, the risk of thrombosis was found to be lowest in IgG-type MGUS compared with IgM- or IgA-type MGUS [28]. The mechanism of VTE is, however, not fully understood, and data regarding prothrombotic coagulation abnormalities in plasma cell disorders other than multiple myeloma (MM) are still limited. One single-center study assessed coagulation abnormalities in various types of plasma cell disorders including MGUS. Factor VIII and von Willebrand factor levels were found to be increased among MGUS cases and the increase was similar to that in patients with MM [17]. Furthermore, there are data to suggest an association between hypercoagulation and progressive neoplastic activity, suggesting that VTE could potentially be a marker of progression toward MM among patients with MGUS [29,30,31]. Future studies are needed to investigate these associations and the potential implications for VTE prophylaxis in the clinical setting.

Regarding structural heart disease and arrhythmias, our matched analyses demonstrated a statistically significant increase in the risk of heart failure, conduction disorders, atrial fibrillation, and aortic and mitral valvular disease, except mitral stenosis, in MGUS patients. There are limited data evaluating these outcomes in MGUS patients. The association between heart failure and MGUS is variable in the literature, with some studies reporting a correlation, while others indicate none [22,23,32]. Some studies have reported cardiac involvement in MGUS secondary to non-amyloidotic monoclonal immunoglobulin light-chain deposits in the myocardium, which can be found in these patients [32,33]. At the cytokine level, proinflammatory cytokines such as osteoprotegerin (OPG) and receptor activator of NF-kB ligand (RANKL) may be involved in cardiac remodeling and the progression of chronic heart failure [34,35,36]. These biomarkers also reflect calcification and are strongly associated with valvular disease and atherosclerotic disease formation in general patient populations [37]. Several studies have reported increased serum levels of OPG and RANKL in MGUS patients too, suggesting that they might play a role in the pathophysiology of cardiovascular disease in this population [37,38]. A high free light-chain (FLC) ratio, which is also seen in MGUS patients, has been reported to correlate with secondary heart failure and severe cardiac involvement in cardiac amyloidosis patients [39,40,41]. In several case reports on a rare type of cardiomyopathy associated with MGUS, treatment with MGUS-directed chemotherapy, such as bortezomib, cyclophosphamide, and dexamethasone, led to a recovery of systolic function in patients [42,43]. These findings may be clinically relevant and suggest that patients with heart failure or cor pulmonale should be screened for plasma cell dyscrasias, such as MGUS. Similarly, they suggest that patients with MGUS should undergo cardiopulmonary testing during follow-up to screen for cardiac involvement using routine cardiac biomarkers, electrocardiograms, and echocardiograms.

Concerning atherosclerotic disease, our univariate analysis found there was a significant increase in PVD and acute MI in the MGUS cohort relative to the non-MGUS cohort in a hospital setting. After we matched our cohorts by age, gender, and race, the acute MI and ischemic stroke/TIA odds were negatively associated with MGUS. However, in the sensitivity analysis that excluded patients with comorbidities, this association lost significance. These findings suggest that in the matched analysis, other comorbidities had a strong positive effect on acute MI and stroke/TIA. This effect likely overshadowed the impact of MGUS, rendering it negatively associated with these outcomes when trying to reach the linear regression line in the analysis. Previous studies in the literature have reported increased risks of myocardial infarction and ischemic stroke. A Danish study showed an increased risk of acute MI, ischemic stroke, PVD, aortic aneurysms, and aortic dissection [22]. Another large Swedish retrospective cohort study reported an increased risk of coronary artery disease and ischemic stroke [10]. The increased risk of atherosclerosis may be linked to clonal hematopoiesis of indeterminate potential (CHIP), which is the presence of an expanded somatic blood cell clone in people who do not have any other hematologic abnormalities [18,19]. Moreover, as mentioned earlier, the upregulation of certain biomarkers like OPG, RANKL, and other inflammatory cytokines like IL-6, as well as immune complex deposition, in MGUS patients may contribute to the accelerated atherosclerosis seen in these patients [34,35,36]. Our findings help expand the literature regarding the cardiovascular outcomes of MGUS and highlight variable results in describing the association of MGUS with atherosclerotic outcomes. The exact cause of these contradictory results is not yet clear, and more research is needed to understand the underlying mechanisms and pathophysiology.

Our study has several strengths that are worth mentioning. First, we assessed MGUS patients in a hospital setting, which is largely neglected in the literature because most patients with MGUS are diagnosed and managed in an outpatient setting. Second, our analyses utilized a large diverse US population of MGUS patients of over 20,000, which is within a reasonable, accurate, and appropriate estimate of MGUS’s prevalence in the generalized and national population. Third, unlike other studies, which were conducted on more homogenous populations, ours included a wide range of different races in the population, which increases the generalizability of our findings. Finally, our study involved matching baseline characteristics and sensitivity analyses that excluded patients with comorbidities, which strengthens the reliability of our assessments of various cardiovascular outcomes. Our study substantially adds to the literature on cardiovascular comorbidities and outcomes among patients with MGUS by describing cardiovascular outcomes in a hospital setting.

There are several limitations of this study. This study was retrospective in nature, and our results may change over time as more data are collected. The NIS database also utilizes ICD codes for classifying many of our comorbidities, including MGUS, and so these data can be prone to coding errors or underreporting, such as an undiagnosed MGUS [44]. Undiagnosed rare comorbidities that were excluded, such as amyloidosis or Waldenstrom’s macroglobulinemia, can affect the results of this study. There is also a lack of detailed clinical data in the NIS database, including information on the subtypes of MGUS and risk stratification (low/intermediate/high risk), which could have affected the outcomes [11,28]. Another limitation is the lack of data regarding the diagnostic workup for patients with MGUS. By definition, MGUS is characterized by <10% bone marrow clonal plasma cells and the absence of lytic lesions in skeletal imaging. However, not all patients suspected of having MGUS undergo bone marrow aspiration and advanced imaging, such as PET/CT scans, as several studies have indicated that approximately 50% of patients diagnosed with MGUS are classified as low-risk, and this workup can be deferred [4]. This could lead to diagnostic bias, as some of these patients might actually have smoldering myeloma, multiple myeloma, or other lymphoproliferative disorders [45]. While our study was able to make independent associations between MGUS and cardiovascular outcomes, a causal association between MGUS and cardiovascular outcomes still remains undetermined [46]. Our study was also prone to selection bias as we focused on inpatient cardiovascular outcomes, whereas a majority of MGUS studies are conducted in an outpatient setting since MGUS presents asymptomatically and usually does not require hospital admission. However, MGUS in an inpatient setting should not be overlooked as diseases that are commonly managed in an outpatient setting may have improved outcomes after hospitalization [47,48]. Larger and long-term prospective studies with standardized MGUS screening tests, such as iStopMM, are needed in the future to determine whether these associations are real, which will help make formal recommendations about screening and primary prevention of cardiovascular disease in MGUS patients [49].

## 5. Conclusions

In conclusion, our study found that patients with MGUS are at an increased risk of a wide spectrum of cardiovascular diseases in a hospital setting. MGUS patients may benefit from cardiovascular screening tools like electrocardiograms and echocardiograms at the hospital as this disease is independently associated with cardiovascular outcomes. Additional studies are needed to corroborate these findings as this will have implications for the monitoring and screening of cardiovascular complications in MGUS patients.

## Figures and Tables

**Table 1:** Baseline characteristics. *—excluding multiple myeloma, amyloidosis, B-cell proliferative lymphomas/disorders, Non-Hodgkin lymphoma, Waldenstrom macroglobulinemia.

Baseline	MGUS (n = 23,435), %	Control (n = 17,334,121), %	*p*-Value
Age	74.0 ± 11.7	57.9 ± 20.3	<0.001
Female	11,352 (48.4)	10,033,318 (57.9)	<0.001
Race			<0.001
White	16,123 (68.8)	11,664,542 (67.3)	
Black	1870 (8.0)	2,626,275 (15.2)	
Hispanic	1367 (5.8)	1,937,970 (11.2)	
Asian/Pacific Islander	520 (2.2)	475,872 (2.7)	
Native American	75 (0.3)	109,652 (0.6)	
Other	480 (2.0)	19,810 (0.1)	
Smoking	7322 (31.2)	3,707,899 (21.4)	<0.001
Hypertension	6215 (26.5)	5,993,378 (34.6)	<0.001
Diabetes mellitus	9186 (39.2)	4,838,241 (27.9)	<0.001
Chronic kidney disease	7866 (33.6)	1,495,853 (8.6)	<0.001
Dialysis dependence	3117 (13.3)	691,122 (4.0)	<0.001
Obesity	4224 (18.0)	2,866,871 (16.5)	<0.001
Dyslipidemia	9174 (39.1)	4,042,393 (23.3)	<0.001
Metabolic syndrome	4427 (18.9)	1,810,488 (10.4)	<0.001
Cancer (all-cause) *	3030 (12.9)	1,408,907 (8.1)	<0.001
Medications			
Antiplatelet	836 (3.6)	446,287 (2.6)	<0.001
Aspirin	4603 (19.6)	2,163,253 (12.5)	<0.001
Oral anticoagulants	3229 (13.8)	1,372,524 (7.9)	<0.001

**Table 2:** Cardiovascular outcomes: CV—cardiovascular; MI—myocardial infarction; VTE—venous thromboembolism; TIA—transient ischemic attack; PVD—peripheral vascular disease.

CV Outcome	MGUS (n = 23,435), %	Control (n = 17,334,121), %	*p*-Value
Heart failure	8631 (36.8)	2,973,087 (17.2)	<0.001
Atrial fibrillation	2333 (10.0)	1,021,056 (5.9)	<0.001
Acute MI	1044 (4.5)	620,304 (3.6)	<0.001
Aortic aneurysm	527 (2.2)	180,457 (1.0)	<0.001
Aortic dissection	21 (0.1)	16,850 (0.1)	0.709
Aortic stenosis	936 (4.0)	285,858 (1.6)	<0.001
Aortic regurgitation	271 (1.2)	81,284 (0.5)	<0.001
Mitral stenosis	62 (0.3)	21,219 (0.1)	<0.001
Mitral regurgitation	830 (3.5)	268,280 (1.5)	<0.001
Conduction disorder	1544 (6.6)	553,692 (3.2)	<0.001
Pericarditis	28 (0.1)	13,855 (0.1)	0.032
PVD	3374 (14.4)	978,262 (5.6)	<0.001
Cor pulmonale	105 (0.4)	37,091 (0.2)	<0.001
VTE	664 (2.8)	281,894 (1.6)	<0.001
Ischemic stroke/TIA	658 (2.8)	457,874 (2.6)	0.113

**Table 3:** Association of monoclonal gammopathy of undetermined significance with cardiovascular outcomes. MI—myocardial infarction; VTE—venous thromboembolism; TIA—transient ischemic attack; PVD—peripheral vascular disease; *—matched by age, gender, and race; ^H^—considered healthy population (absence of diabetes mellitus, prior myocardial infarction, chronic kidney disease (stages 3–5), dialysis, hypertension, obesity, metabolic syndrome, cancer (all-cause), smoking, dyslipidemia, and aspirin, antiplatelet, and oral anticoagulant use).

Cardiovascular Outcome	OR *	CI	*p*-Value	OR ^H^	CI	*p*-Value
Heart failure	1.46	1.40–1.51	<0.001	1.34	1.17–1.54	<0.001
Atrial fibrillation	1.14	1.09–1.19	<0.001	1.45	1.25–1.69	<0.001
Acute MI	0.88	0.81–0.96	0.004	1.00	0.75–1.34	0.993
VTE	1.31	1.17–1.47	<0.001	1.84	1.31–2.57	<0.001
Ischemic stroke/TIA	0.66	0.60–0.73	<0.0001	0.99	0.74–1.33	0.943
Aortic aneurysm	1.13	0.99–1.28	0.063	1.00	0.64–1.55	0.998
Aortic dissection	0.65	0.39–1.08	0.098	1.69	0.24–11.98	0.601
Aortic stenosis	1.13	1.03–1.24	0.013	1.51	1.05–2.17	0.026
Aortic regurgitation	1.19	0.99–1.42	0.05	2.30	1.15–4.59	0.018
Mitral stenosis	1.32	0.90–1.93	0.15	3.38	0.62–18.45	0.160
Mitral regurgitation	1.42	1.28–1.58	<0.001	2.06	1.40–3.01	<0.001
Conduction disorder	1.10	1.02–1.18	0.017	1.20	0.94–1.53	0.137
Pericarditis	1.22	0.70–2.11	0.484	2.03	0.62–6.65	0.244
Cor pulmonale	1.59	1.17–2.17	0.003	5.07	1.02–25.14	0.047
PVD	1.36	1.26–1.48	<0.001	2.40	1.95–2.95	<0.001

## Data Availability

While the datasets in the Healthcare Cost and Utilization Project are public and available through the HCUP Central Distributor, data availability is restricted to people approved by the Agency for Healthcare Research and Quality to purchase the data. To receive approval from the Agency of Healthcare Research and Quality to purchase the data, applicants must complete the HCUP Data Use Agreement Training and sign an HCUP DUA. The datasets can be requested when accessing the online HCUP Central Distributor. Information regarding purchasing this publicly available database can be found at https://hcup-us.ahrq.gov/tech_assist/centdist.jsp (accessed on 1 September 2022), and instructions may be found at https://hcup-us.ahrq.gov/tech_assist/faq.jsp#PurchasingFAQ (accessed on 1 September 2022).

## Supplementary Materials

The following supporting information can be downloaded at: https://www.mdpi.com/article/10.3390/curroncol31080331/s1, Table S1: Various cancer subtypes at baseline.
