# Supplementary material for: Monoclonal Gammopathy of Undetermined Significance and Associated Cardiovascular Outcomes in a Hospital Setting—A Fresh Perspective
Source: Curr Oncol. 2024 Aug 1;31(8):4432–42. doi: 10.3390/curroncol31080331 (PMC11352401; doi:10.3390/curroncol31080331)
Supplement: Supplementary file 1 [file curroncol-31-00331-s001.zip › curroncol-3088665-supplementary.pdf]

Supplemental Table S1. Various cancer subtypes at baseline.

	MGUS (n=23435), %	Control (n=17334121), %
Gastrointestinal cancer (C15-C25)	387 (1.7)	314034 (1.8)
Lung cancer (C34)	416 (1.8)	232498 (1.3)
Breast cancer (C50)	131 (0.6)	98778 (0.6)
Gynecologic cancer (C51-C57)	85 (0.4)	84860 (0.5)
Prostate cancer (C61)	339 (1.4)	114321 (0.7)
Urinary cancer (C64-C67)	229 (1.0)	101730 (0.6)
Skin cancer (C43-C44)	46 (0.2)	17147 (0.1)
Hematologic (C81-C96) *	2642 (2.7)	146711 (0.8)

Numbers in parenthesis refer to ICD-10 codes. * excluding people with amyloid disease, lymphoma, or multiple myeloma
